# 108. Efficacy of Dalbavancin Compared to Standard of Care for the Treatment of Osteomyelitis: A Retrospective Study

**DOI:** 10.1093/ofid/ofab466.108

**Published:** 2021-12-04

**Authors:** Alexander Cain, Derek N Bremmer, Dustin R Carr, Carley Buchanan, Max Jacobs, Thomas L Walsh, Matthew Moffa, Nathan Shively, Tamara Trienski

**Affiliations:** 1 Allegheny General Hospital, Pittsburgh, Pennsylvania; 2 Allegheny Health Network, pittsburgh, PA

## Abstract

**Background:**

Preliminary data suggest that the efficacy of dalbavancin, a long-acting lipoglycopeptide, may be similar to current standard of care (SoC) treatment options for osteomyelitis, and may be associated with fewer treatment related adverse events. This study assessed the incidence of treatment failure in patients receiving either dalbavancin or SoC for the treatment of osteomyelitis.

**Methods:**

This was a multi-center, retrospective, observational cohort study of adult patients diagnosed with osteomyelitis. Patients were matched 1:2 to either dalbavancin (1500 mg infused intravenously on days 1 and 8) or SoC for osteomyelitis (oral or intravenous antibiotics) by Charlson Comorbidity Index, site of infection, and causative pathogen. The primary objective was to determine the incidence of treatment failure after a one-year follow-up period. Secondary objectives included hospital length of stay (LOS), infection related one-year readmission rates, and treatment related adverse events.

**Results:**

A total of 132 patients were matched to receive dalbavancin (n = 42) or SoC (n = 90). Baseline characteristics were similar between the two treatment groups. The majority of patients had lower extremity osteomyelitis (76.2% vs 73.3%) with an etiology of diabetic foot infection (45.2% vs 46.7%) in the dalbavancin and SoC groups, respectively. Treatment failure was similar between those who received dalbavancin and SoC (21.4% vs 23.3%, p = 0.808). Patients who received dalbavancin had a significantly shorter hospital LOS compared to patients who received SoC regimens (5.2 days vs 7.2 days, p = 0.013). There was no difference in the rates of infection related readmissions between the dalbavancin and the SoC group (31% vs 31.1%, p = 0.985). Peripherally inserted central catheter line related complications were reported in 17.8% of patients in the SoC group, however the lower incidence of overall adverse events in the dalbavancin group was not significantly different than the SoC group (21.4% vs 36.7%, p = 0.08).

**Conclusion:**

Dalbavancin administered as a two-dose regimen is a safe and effective option for the treatment of osteomyelitis

**Disclosures:**

**Dustin R. Carr, PharmD, BCPS, BCIDP, AAHIVP**, **Merck** (Speaker's Bureau) **Thomas L. Walsh, MD**, **Accelerate Diagnostics** (Other Financial or Material Support, speaking fees)

